# Supplementary material for: Usefulness of Mycobacterium tuberculosis-polymerase chain reaction with bronchial washing samples in predicting discontinuation of airborne infection isolation in patients hospitalized with suspected pulmonary tuberculosis
Source: PLoS One. 2022 Dec 30;17(12):e0279256. doi: 10.1371/journal.pone.0279256 (PMC9803188; doi:10.1371/journal.pone.0279256)
Supplement: S1 Table — (DOCX) [file pone.0279256.s001.docx]

**Table S1. Diagnostic yield of MTB-PCR using bronchoscopy samples considering AFB culture as the gold standard in patients with negative sputum AFB smear and MTB-PCR results**

|  | Culture  (+) | Culture  (-) | Total | Sensitivity  % | Specificity  % | PPV  % | NPV  % |
| --- | --- | --- | --- | --- | --- | --- | --- |
| MTB-PCR  (+) | 11 | 5 | 16 | 61.1 (35.7–82.7) | 95.5 (89.9–98.5) | 68.7 (46.4–84.3) | 93.9 (89.6–96.5) |
| MTB-PCR  (-) | 7 | 108 | 115 |  |  |  |  |
| Total | 18 | 113 | 131 |  |  |  |  |

Data are presented as numbers. (+) positive; (−) negative.

MTB, *Mycobacterium tuberculosis*; PCR, polymerase chain reaction; AFB, acid-fast bacilli; PPV, positive predictive value; NPV, negative predictive value
