# Supplementary material for: Usefulness of Mycobacterium tuberculosis-polymerase chain reaction with bronchial washing samples in predicting discontinuation of airborne infection isolation in patients hospitalized with suspected pulmonary tuberculosis
Source: PLoS One. 2022 Dec 30;17(12):e0279256. doi: 10.1371/journal.pone.0279256 (PMC9803188; doi:10.1371/journal.pone.0279256)
Supplement: S2 Table — (DOCX) [file pone.0279256.s002.docx]

**Table S2. Subgroup analysis of patients with positive MTB-PCR results**

| **Variables** | **Positive results of culture (n = 22)** | **Negative results of culture (n = 5)** | **P value** |
| --- | --- | --- | --- |
| Age, years | 74.0 (49.0 – 76.7) | 67.0 (64.0 – 77.0) | 0.975 |
| Sex, male | 11 (50.0%) | 5 (100%) | 0.060 |
| Hypertension | 8 (36.4%) | 3 (60.0%) | 0.370 |
| Diabetes mellitus | 5 (22.7%) | 3 (60.0%) | 0.136 |
| TB history | 1 (4.5%) | 1 (20.0%) | 0.342 |
| COPD | 1 (4.5%) | 0 (0%) | 1.000 |
| Old cerebrovascular disease | 2 (9.1%) | 1 (20.0%) | 0.474 |
| Initial symptoms |  |  |  |
| Cough | 6 (27.3%) | 3 (60.0%) | 0.295 |
| Sputum | 3 (13.6%) | 3 (60.0%) | 0.056 |
| Fever | 5 (22.7%) | 2 (40.0%) | 0.580 |
| Dyspnea | 8 (36.4%) | 4 (80.0%) | 0.139 |
| Hemoptysis | 1 (4.5%) | 1 (20.0%) | 0.342 |
| Diagnostic evaluation |  |  |  |
| Sputum AFB smear, positive | 6 (27.3%) | 0 (0%) | 0.216 |
| AFB smear using washing samples, positive | 8 (36.4%) | 0 (0%) | 0.280 |
| Radiologic findings |  |  |  |
| Nodules | 21 (95.5%) | 3 (60.0%) | 0.079 |
| Consolidations | 15 (68.2%) | 2 (40.0%) | 0.326 |
| Cavities | 7 (31.8%) | 1 (20.0%) | 1.000 |
| Ground-glass opacities | 1 (4.5%) | 3 (60.0%) | 0.013 |
| Bronchiectasis | 1 (4.5%) | 2 (40.0%) | 0.079 |
| Old tuberculosis | 7 (31.8%) | 3 (60.0%) | 0.326 |
| Upper lobe involvement | 22 (100%) | 4 (80.0%) | 0.185 |

Data are presented as the median (interquartile range) or number (%).

PCR, polymerase chain reaction; COPD, chronic obstructive pulmonary disease; AFB, acid-fast bacilli; MTB, *Mycobacterium tuberculosis*
