# Supplementary material for: Usefulness of Mycobacterium tuberculosis-polymerase chain reaction with bronchial washing samples in predicting discontinuation of airborne infection isolation in patients hospitalized with suspected pulmonary tuberculosis
Source: PLoS One. 2022 Dec 30;17(12):e0279256. doi: 10.1371/journal.pone.0279256 (PMC9803188; doi:10.1371/journal.pone.0279256)
Supplement: Attachment — Submitted filename: Response to Reviewers.docx [file pone.0279256.s004.docx]

**PONE-D-21-35876**

**Usefulness of Mycobacterium tuberculosis-polymerase chain reaction with bronchial washing samples in predicting discontinuation of airborne infection isolation in patients hospitalized with suspected pulmonary tuberculosis**

PLOS ONE

**Responses to reviewers**

Reviewer #1

**Q1. Lines 73 and 74. In the exclusion criteria, I understand that patients who were undergoing treatment for tuberculosis were excluded from the study. But why patients who were only diagnosed clinically or histologically were excluded?**

**A1. Thank you for your comments.**

Three patients with clinically diagnosed tuberculosis were excluded from this study. Despite being clinically diagnosed with TB, there was a limitation in evaluating the adequacy of air isolation in patients who showed negative results in all tests, including bronchoscopy.

One patient with histologically diagnosed TB was also excluded from this study. TB-PCR, TB-culture, and AFB/S tests were negative in the patient. Histologically, only granulation tissue was observed, but tissue TB-PCR was negative. Because it was unclear whether the patient had tuberculosis, we excluded the patient from this study.

**Q2. Line 160. Where it says Of 166 patients, 11 (non-TB group: 6; TB group: 3) it should say Of 166 patients, 9 (non-TB group: 6; TB group: 3)**

**A2. Thank you for your comments.** We changed the manuscript as your recommendation. [See line 160 on page 9]

**Q3. Why are in table 1 26 positive cultures for M. tuberculosis and in table 2 are 30 positive cultures?**

**A3. Thank you for your comment.** As shown in Table 1, positive culture results were observed for 21 patients in sputum and for 26 patients in washing samples. Of these 17 patients had positive results both in sputum and washing samples. Thus, total 30 patients had positive cultures either in sputum or washing samples. We modified the manuscript of results section. [See line 164-167 on page 10]

**Q4. Line 176. Where it says Of 136 patients with negative results it should say Of 139 patients with negative results**

**A4. Thank you for your valuable comment.** We changed the manuscript as your comment. [See line 194 on page 12]

**Q5. In lines 181 and 182 the name of table 2 instead of being**

Table 2. Diagnostic yield of MTB-PCR using bronchoscopy samples considering AFB culture as the gold standard should be Table 2. Diagnostic yield of MTB-PCR using bronchoscopy samples considering AFB culture as the gold standard.

A5. The suggested sentence is the same as the existing sentence of title of table 2. [See line 184-185 on page 11]

**Q6. In line 191 where it says A total of 139 patients were negative for TB it should say A total of 131 patients were negative for TB**

**A6. Thank you for your valuable comment**. This sentence was deleted because its meaning was unclear. Instead, we provided a detailed description of predictive accuracy. [See line 194-195 on page 12]

**Q7. In line 192 If we take into account that there were 131 negative cultures for M.tuberculosis and 139 negative MTB-PCR, the predictive accuracy would be 94.2%.**

**A7. Thank you for your valuable comment.** As we mentioned in the method section, the prediction of AII discontinuation is calculated as the percentage of patients with three consecutive negative AFB smear test results (n = 7) or were established not to have pulmonary TB (n = 131) out of patients with negative MTB-PCR results using bronchial washing samples (n = 139) [$\frac{7+131}{139}\times100 (\%)$]. Therefore, the prediction of the discontinuation of AII is 99.2%. We also modified abstract in the result section. [See line 194-195 on page 12]

**Q8. The authors explain very well the characteristics of the 8 patients who showed negative results for MTB-PCR using sputum and washing samples and positive culture for M.tuberculosis. But it is also important to explain the characteristics of the 5 patients who showed a positive MTB-PCR result and a negative culture for M.tuberculosis.**

**A8. Thank you for your valuable comments.** A subgroup analysis was conducted for 27 patients with positive MTB-PCR results as your recommendation. Five of the 27 patients with positive MTB-PCR results in washing samples had negative TB cultures. There were more ground-glass opacities on chest CT in culture-negative patients than in culture-positive patients (1 [4.5%] vs. 3 [60.0%]; P = 0.013). There were no significant differences between the two groups in terms of other clinical variables. These results have been added to Table S1 and the results section. [See line 216-218 on page 14]
