# Supplementary material for: Usefulness of Mycobacterium tuberculosis-polymerase chain reaction with bronchial washing samples in predicting discontinuation of airborne infection isolation in patients hospitalized with suspected pulmonary tuberculosis
Source: PLoS One. 2022 Dec 30;17(12):e0279256. doi: 10.1371/journal.pone.0279256 (PMC9803188; doi:10.1371/journal.pone.0279256)
Supplement: Attachment — Submitted filename: Response to reviewers.docx [file pone.0279256.s005.docx]

**PONE-D-21-35876R1**

**Usefulness of Mycobacterium tuberculosis-polymerase chain reaction with bronchial washing samples in predicting discontinuation of airborne infection isolation in patients hospitalized with suspected pulmonary tuberculosis**

PLOS ONE

Reviewer #2: I have read the V1 and R1 version of the article and the other reviewer’s comment to V1. This is my first revision of the article. I have some additional comments that in my opinion may improve the quality of the paper.

Q1) I think it would be of interest to know how many patients with suspected TB were included because of AFB and MTB-PCR negative results in sputum smear, and how many because they could not expectorate. Presenting data of patients with MTB-PCR negative in sputum, but positive only after bronchoscopy would be of interest, indicating the sensitivity of MTB-PCR on bronchoscopy in patients with negative results in sputum.

A1) Thank you for your comments. There were 131 patients with negative results for both sputum AFB smears and sputum TB-PCR tests. Sixteen were positive in the MTB-PCR test using washing specimens and eighteen were positive for MTB culture. Of 16 patients who had positive results on MTB-PCR using washing specimens, 11 showed positive MTB culture. Of 115 patients with negative results on MTB-PCR using washing specimens, 7 showed positive MTB culture. MTB-PCR using washing specimens showed 61.1% sensitivity, 95.5% specificity, 68.7% positive predictive value, and 93.9% negative predictive value in patients with negative sputum AFB smear and MTB-PCR results. We presented in table S1. (See line 191-198 on page 12)

Q2) It is somehow surprising the six patients with positive AFB in smear were finally diagnosed of pulmonary TB only after bronchoscopy. Was MTB-PCR performed on the positive AFB sputum sample and were in all the six patients negative? (MTB-PCR in AFB+ sputum samples has a very high sensitivity close to 100%)

A2) Thank you for your comments. The sputum AFB smear test was positive in nine of the patients enrolled in this study. Sputum TB-PCR was performed by six of them, but all of them were negative. AFB smears and TB-PCR results were positive in four of these six patients. NTM was cultured for the other two patients. AFB smear and TB-PCR tests performed on washing samples were positive for two out of three patients who did not undergo sputum TB-PCR. All tests on the remaining patient's washing specimen were negative. Sputum MTB-PCR has a very high sensitivity if AFB smear is positive in a sputum sample of a patient with pulmonary tuberculosis, as you mentioned in your comments. There were four patients in this study who had positive sputum AFB smears, but negative sputum MTB-PCRs, and tuberculosis was diagnosed through washing samples. There may have been errors in the sputum MTB-PCR tests or insufficient samples of sputum MTB-PCR have been collected. We added this limitation in the limitation section. (See line 274-278 on page 16-17)

Other minor comments:

Q3) It is difficult for me to understand inclusion criteria of the initial review of 216 patients:

Fig-1 and page 9, lines 140-145. The authors state that inclusion criteria were “patients under AII who underwent fiberoptic bronchoscopy for pulmonary TB, because they were unable to expectorate or because TB could not be ruled out based on the results of initial AFB smear and MTB-PCR”. So it is difficult for me to understand why among the initial 216 patients (“hospitalized patients under AII owing to suspected pulmonary TB who underwent bronchoscopy”) are included 16 patients already under treatment for TB; 8 patients with positive MTB-PCR in sputum or 3 patients with clinically diagnosed TB (they were already with a TB dg at the time performing the bronchoscopy).

A3) Thank you for your valuable comments. To identify patients under AII who underwent fiberoptic bronchoscopy for pulmonary TB, because they were unable to expectorate or because TB could not be ruled out based on the results of initial AFB smear and MTB-PCR, the following patients were screened at first: patients under AII who underwent fiberoptic bronchoscopy during the study period. 216 patients were screened, and 50 were excluded based on the exclusion criteria. To clarify the meaning of figure 1, we changed it. (See line 72-73 on page 5 and figure 1)

Q4) Page 4. Line 57. Interval 8 to 24 hours (not just 8h) according to ref #3.

A4) Thank you for your valuable comment. We change the manuscript as your recommendation. (See line 57 on page 4)

Q5) Although I’m not an English native speaker; I think there are some grammatical errors: page 6; line-109: results of a MTB-PCR; line: 111: if TB was ruled out...

A5) Thank you for your comment. We changed the manuscript as your comment. (See line 110 and 112 on page 6)
